# The role of atherectomy in the treatment of lower extremity peripheral artery disease

**DOI:** 10.1186/1471-2482-12-S1-S13

**Published:** 2012-11-15

**Authors:** Anna Franzone, Marco Ferrone, Giuseppe Carotenuto, Andreina Carbone, Laura Scudiero, Federica Serino, Fernando Scudiero, Raffaele Izzo, Raffaele Piccolo, Savio Saviano, Bruno Amato, Cinzia Perrino, Bruno Trimarco, Giovanni Esposito

**Affiliations:** 1 Department of Clinical Medicine, Cardiovascular and Immunological Sciences, Federico II University, Via S. Pansini 5, 80131, Naples, Italy; 2 Department of General, Geriatric, Oncologic Surgery and Advanced Technologies, Federico II University, Via S. Pansini 5, 80131, Naples, Italy

## Abstract

**Background:**

The incidence of lower extremity peripheral artery disease (LE-PAD) continues to increase and associated morbidity remains high. Despite the significant development of percutaneous revascularization strategies, over the past decade, LE-PAD still represents a unique challenge for interventional cardiologists and vascular surgeons.

**Method:**

Typical features of atherosclerosis that affects peripheral vascular bed (diffuse nature, poor distal runoff, critical limb ischemia, chronic total occlusion) contribute to the disappointing results of traditional percutaneous transluminal angioplasty (PTA). New technologies have been developed in attempt to improve the safety and effectiveness of percutaneous revascularization. Among these, atherectomy, debulking and removing atherosclerotic plaque, offers the potential advantage of eliminating stretch on arterial walls and reducing rates of restenosis.

**Conclusions:**

This review summarizes the features and the current applications of new debulking devices.

## Introduction

Peripheral artery disease (PAD) is a common manifestation of systemic atherosclerosis causing a chronic, slowly developing, narrowing of the arteries. Lower extremity peripheral artery disease (LE-PAD) typically affects lower limbs with a frequency that is strongly age-related (~20% of the population older than 55) [1]. Clinical manifestations vary from intermittent claudication (pain in the calves while walking that goes away with rest) to critical limb ischemia (CLI), gangrene and, ultimately, limb loss. A significant proportion of patients with PAD have an increased risk of stroke or myocardial infarction [2]. The strong relationship between PAD and coronary heart disease (CHD) is the consequence of common etiology and pathophysiology and has important prognostic implications. Hypoechoic, vulnerable, rupture-prone, plaques in the femoral arteries represent a marker of cardiovascular risk and are associated to higher incidence of major cardiovascular events [3]. Moreover, successful revascularization of lower limbs by percutaneous transluminal angioplasty (PTA) improves functional status and quality of life and, interestingly, reduces the occurrence of future atherothrombotic events [4]. A number of pharmacological and invasive strategies have been developed over the last decades to improve the clinical outcomes of patients affected by LE-PAD. A “non-interventional” management is the first choice for patients with intermittent claudication: smoking cessation, nutritional counseling, exercise program, strict control of diabetes and hypertension and medication (antiplatelet agent and statins) [5,6]. Mild to moderate benefits have been associated to other pharmacological agents such as pentoxyphylline and cilostazol that resulted effective in increasing walking distance [5]; on the other hand, the intensification of antiplatelet therapy by adding vorapaxar, a novel antiplatelet agent that inhibits cellular actions of thrombin, has shown to reduce atherothrombotic events in patients with PAD [7]. In the past, bypass surgery was the standard of care for patients with severe claudication and CLI [8]. Recently, technological advances favoured a significant evolution of percutaneous revascularization therapies that now can be offered as treatment options less invasive than traditional surgery. The optimal endovascular therapy is not well established: there are several options including percutaneous transluminal angioplasty (PTA), stents, drug-coated balloons, crioplasty, percutaneous thrombectomy (rheolytic [9] and aspiration thrombectomy) and atherectomy [10,11]. In particular, the promise of atherectomy is to overcome the limitations and complications of traditional angioplasty such as dissection, elastic recoil, and disruption of the internal elastic lamina, resulting in overwhelming neo-intima and smooth muscle cell proliferation [12]. Unlike balloons and stents, which push plaque into the vessel wall, atherectomy offers the ability to debulk the plaque burden within the vessel [13]. The concept of directional and laser atherectomy is not new and was investigated extensively in the coronary arteries and saphenous vein bypass graft [14]. Despite previous coronary artery data regarding atherectomy may not translate to the peripheral vascular bed because of peculiar dynamic forces acting on these arteries, increased awareness about percutaneous treatment of LE-PAD favoured the development of new debulking devices. Various atherectomy methods are available including plaque excision (directional) atherectomy, laser atheroablation, rotational aspiration/atherectomy and orbital atherectomy. Although data from randomized clinical trials are lacking, this review summarizes the current approach to peripheral atherectomy based on evidences from multicenter prospective registries and describes the mechanism of action of currently available atherectomy devices.

### Anatomical features

Atherosclerosis affecting lower limbs has some specific features: diffuse involvement, superimposed calcification, high incidence of progression to total occlusion, large plaque burden [15]. As our understanding of the complexity of atherosclerotic occlusive disease is evolving (through enhanced use of imaging techniques and growing evidence about molecular mechanisms), new technologies and techniques have been developed to better treat LE-PAD. Femoropopliteal artery disease, in particular, is responsible for the majority of cases of symptomatic LE-PAD. The superficial femoral artery (SFA) is one of the most dynamic vessel of the body and, for this reason, the site of frequent failure of percutaneous strategies. The SFA undergoes many biomechanical stresses such as torsion, compression, flexion and extension by large muscular groups. These factors contribute to make endovascular treatment of this vascular bed especially challenging and are responsible of poor long-term outcomes after percutaneous intervention. Although PTA results effective for the treatment of focal iliac stenosis, pooled primary patency rate following femoropopliteal PTA is of 77% at 1 year, 61% at 3 years, and 55% at 5 years [16]. Factors affecting the long-term patency rates of PTA alone are disease severity, lesion length, poor runoff, diabetes, critical limb ischemia and clinical presentation [17]. In this vascular bed, primary arterial patency is poor with stenting too, despite high procedural acute success. Stent fractures at site of excessive movement (SFA and popliteal arteries) are frequent [18]. Moreover, ultrasound evidence of binary restenosis ranges from 8% to 40% between 6 and 24 months [19]. Procedural success rates have been historically lower in the setting of chronic total occlusion that account for up to 20-40% of patients with LE-PAD undergoing percutaneous treatment [20]. In addition, a number of potential complications (in particular, distal embolization, plaque shift and perforation) may contribute to disappointing results of percutaneous approach to complex infrainguinal disease. Atherectomy, by debulking and removing atherosclerotic plaque cutting or pulverizing atheroma, seems to be a favourable option to increase the spectrum of treatable femoropopliteal lesions such as ostial disease (involving profunda femoral artery), densely calcified plaque, diffuse disease.

### Directional atherectomy

The SilverHawk plaque excision system (ev3 Inc., Plymouth, MN) consists of two major components: a low-profile catheter and a palm-sized drive unit with on/off thumb switch. They are packaged separately, but used together during the atherectomy procedure. The device is a monorail exchange system over a 0.014 guidewire that is currently available in 7 differents diameter and catheter lengths to allow treatment of femoral, popliteal, tibial, and even pedal vessels [21]. It contains a carbide cutter disc with variable height and rotates at speeds of 8,000 rpm. With the blade spinning, the catheter is slowly advanced across the lesion and the excissed tissue is captured and stored in the tip of device. The cutting sequence can be repeated as many time as necessary to achieve the desired degree of plaque excision. It can be used without the adjunctive use of balloon angioplasty or stents. Significant debulking of the lesion can be achieved without the barotrauma associated with the previous directional atherectomy catheter that uses a balloon opposite the cutting blade to maximize plaque removal [22]. The major indications for the SilverHawk device are focal eccentric lesions, bifurcation lesions of the infrainguinal arteries including the common femoral artery, bypass anastomotic lesions, and long diffuse femoropopliteal lesions including chronic total occlusions. Unfortunately, there have been no randomized clinical trials using this device comparing it to angioplasty. Available data come from several registries or single-centre studies [23,24]. The largest, nonrandomized registry, TALON (Treating Peripherals with Silver Hawk Outcome Collections) showed excellent procedural success rates of 97.6% and < 50% residual stenosis achieved in 94.7% of lesions; adjunctive angioplasty was used in 21.7%, and stents were used in 6.3% of the patients. The overall 12-month freedom from target lesion revascularization (TLR) rate was 80% [25]. The SilverHawk plaque excision system performs ideally in heavily calcified femoropopliteal lesions; occlusions should be predilated with an undersized balloon to ensure that the wire crosses intraluminally. There are no data supporting the use of this system in the setting of long lesion (>15 cm) and it should be avoided when subintimal crossing is involved [26]. Table 1.

**Table 1:** Registries of patients treated with contremporary directional atherectomy

	TALON	McKinsey et al	Zeller et al
Study type	Observational, nonrandomized, multicenter registry	Prospective study	Prospective, nonrandomized, single-center study

Patients/Lesions	728/1,517	275/579	84/131

Lesion location	SFA/below-the-knee	Infrainguinal	SFA/popliteal

Average lesion length	8.4 cm	SFA, 9.16 cm; popliteal, 3.77 cm; tibial, 4.64 cm	9–10.6 cm

Lesion characteristics	De novo	De novo	Group 1, 34%;a group 2, 33%;b group 3, 33%c

Stand-alone treatment	----	64.8%	----

Adjunctive balloon angioplasty	21.7%	24.3%	59%

Adjunctive stenting	6.3%	7.5%	6%

Primary patency (by duplex) at 12 months	----	62.2%	Group 1, 84%;a group 2, 54%;b group 3, 54%c

18-month primary patency	----	52.7%	Group 1, 73%;a group 2, 42%;b group 3, 49%c

TLR at 12 months	80%	----	Group 1, 16%;a group 2, 44%;b group 3, 47%c

TLR at 18 months	----	----	Group 1, 22%;a group 2, 56%;b group 3, 49%c

Claudication	----	36.7%	----

CLI	----	63.3%	----

Device-related SAEs	5.3%	4.1%	3.8%

aGroup 1 was de novo lesions.

bGroup 2 was native vessel restenosis.

cGroup 3 was in-stent restenosis.

### Rotational atherectomy

The Jetstream G2 (Pathway Medical Technologies) is a rotational aspiration atherectomy device, uniquely combining rotablation with aspiration capability. The device consists of 2 primary components: a sterile, single use unit consisting of an electrically driven, differentially cutting, aspirating, expandable catheter with a control pod assembly, and a console, with 2 peristaltic aspiration pumps for aspiration and infusion. Isosmolar saline solution is attached to the proximal end of the catheter using 2 dedicated lines. The removed, potentially embolic, material is aspirated at the treatment site, via ports in the fluted tip, into the catheter lumen, and transported to a collection bag located on the device console [27]. The fluted tip rotates at approximately 55,000 rpm with a delivery system that is 8 F compatible and uses a 0.014-inch guidewire. The device is designed to treat the wide spectrum of disease found in patients with LE-PAD, including hard and soft plaque, calcium, thrombus, and fibrotic lesions. Based on limited data set, the Pathway system appears to be effective in treating SFA atherosclerotic disease, including cases with the presence of significant calcification. In a multicenter registry using the first-generation Pathway device, 172 patients with 210 lesions in nine European centers were treated; the mean lesion length was 35 mm with moderate to high calcium. The primary endpoint was freedom from device-related serious adverse events (SAEs) at 6 months. TLR at 6 and 12 months was 13% and 26%, respectively. The ABI (Ankle- Brachial Index) increased from 0.59 ± 0.21 at baseline to 0.77 ± 0.26 and 0.82 ± 0.26 (P < .05) at 6 and 12 months, respectively [27]. TRUE (Tissue Removal by Ultrasound Evaluation) study evaluated the debulking properties of this device by analyzing changes in the plaque volume and composition and vessel size using intravascular ultrasound (IVUS) and virtual histology (VH). Freedom from target lesion revascularization (TLR) at 6 and 12 months was also evaluated. In this study atherectomy with the Jetstream G2 system resulted in substantial plaque reduction by removing fibrotic and fibro-fatty plaque [28]. Furthermore, there were no major complications during the procedure or index hospitalization, and an acceptable TLR rate was observed after a 1-year follow-up. Table 2.

**Table 2:** Registries of patients treated with rotational and orbital atherectomy

	Pathway System		Diamondback 360° OAS (CSI)	
Study	Pilot study	Multicenter, prospective registry	OASIS trial	PAD II

Patients/lesions	15/15	172/210	124/201	66/86

Lesion location	47% SFA	64% SFA	94% popliteal and tibial	Reference vessel diameter of 1.5–4 mm

Mean lesion length	61 mm	35 mm	30 mm	35 mm

Calcified	----	----	55%	----

Chronic total occlusion	----	----	12%	----

Stand-alone treatment	6 (40%)	33%	58.2%	39.5%

Adjunctive balloon angioplasty	7 (47%)	59%	39.3%	60.5%

Adjunctive stenting	2 (13%)	7%	2.50%	----

Primary patency (by duplex) at 6 months	73%	----	----	----

TLR at 6 months	0%	13%	2.4%	13.6%

TLR at 12 months	----	26%	----	----

Preprocedure ABI	0.54 ± 0.3	0.59 ± 0.2	0.68 ± 0.2	----

ABI at 6 months	0.81 ± 0.2	0.77 ± 0.3	0.82 ± 0.1	----

ABI at 12 months	----	0.82 ± 0.3	----	----

MAEsa at 1 month	----	----	4 (3.2%)	----

MAEsa at 6 months		15%	13 (10.4%)	37.9%

Device-related SAEs			2.9%	6%

*aDeath*, *myocardial infarction*, *amputation*, *or repeat revascularization.*

### Orbital atherectomy

Diamondback 360° Orbital Atherectomy System, OAS, (Cardiovascular Systems, Inc. [CSI], St. Paul, MN) is an orbital atherectomy system that consists of an eccentric, diamond-grit-coated, abrasive crown that creates an ablative surface proportional to the displaced radius of the crown through centrifugal force when the device is rotated at various speeds [29]. It can create a lumen that is > 1.75 times the crossing profile depending on the size of the grit and the eccentricity of the offset. The greater the speed of the crown, the larger the arc of debulking is and, ultimately, the resultant lumen size [30]. The centrifugal force and the differential sanding allow the removing of thin layer of the calcific plaque to each pass of the device while the differential sanding discriminates between the calcific or fibrocalcific lesion and the normal arterial wall thanks to the elastic compliance of the arterial tissue adjacent to the plaque. OAS is a system which includes a low profile catheter with an eccentrically mounted diamond-coated crown, a control handle with control knob, a flexible drive shaft, and a protective sheath. The mechanism of OAS is based on the rotation of the flexible drive shaft and of the crown over a guide wire (ViperWire Advance). The guide wire operates in this way as the rail on which the catheter rotates, while the sheat covers the drive shaft and allows the delivery of saline solution and medications in the treatment area. The movement and the rotating speed of the crown is managed through the external control unit. The control unit is the user interface and regulates air pressure to drive the speed turbine located in the control handle rotating the drive shaft and the crown to speed up to 20.000 rpm. A constant flow of saline solution is delivered by a roller pump in the control unit which lubrificates the device and helps to flush the artery. The high speed rotation, together with the diamond coated crown modifies the morphology of the fibrocalcific plaque and acts as an orbital atherectomy sanding the profile of the lesion at each pass of the device. This device uniquely delivers 360° of plaque removal and may be effective in calcified plaque [31]. The off-centered shape of the crown allows for continuous blood flow around the device, which allows particles to move downstream, and reduces localized heating of the vessel [30]. OASIS (Orbital Atherectomy System for the Treatment of Peripheral Vascular Stenosis), a nonrandomized prospective Investigational Device Exemption study, enrolled 124 patients (202 lesions) at 17 sites in the United States between January 2006 and January 2007; procedural success (achievement of < 30% residual diameter stenosis) was met in 90.1% of lesions. Orbital atherectomy was used alone in 57.7% of lesions, with adjunctive therapy (PTA and/or stenting) used in 42.3%. Symptom- driven TLR at 6 months was 2.4%; the mean Rutherford categories were 3 ± 1.3 at baseline and 1.2 ± 1.5 at 6 months. Interestingly, 14% of the patients demonstrated worse ABI at the end of 30 days compared to baseline. This detriment in ABI may be secondary to procedural complications due to hemolysis or distal embolization [32]. Obviously, this procedure can imply different intra-procedurals complications. Slow flow is a common complication in every kind of atherectomy procedure for the liberation of debris clogging microcirculation. Several testing in vivo were conducted to evaluate the impact of sanding and of liberation of debris during the orbital atherectomy. Both the classic and the solid crown were tested in this sense, and both showed to generate particles of a diameter smaller than 9.5 micron which is the diameter of the capillars. Therefore the most of the particles can be flushed through the capillary bed. The effect of the generation of the particles during the procedure, however, can be minimized ensuring proper orbital sanding technique and using vasodilators. Orbital sanding speed should be increased gradually and for short period of time, while vasodilators drugs should be used at the beginning of the procedure and during intervals helping the dispersion of particles. Hemolysis is another complication associated to high-speed orbital atherectomy. Nevertheless studies in orbital sanding system showed a minimal and transient haemolysis. OASIS trial did not show clinical significant hemolysis associated to OAS atherectomy, and even if laboratory markers did fluctuated after the procedure, this changes were transient and resolved spontaneously. Arterial spasm occurs during the procedure in the site corresponding the orbital atherectomy or at site just adiacent. Pain can occur in the site of the treatment and it is most likely due to the activation of the adventitial nerve receptors as the media and intima are not innervated. Obviously the choise of proper crown dimension and limiting the orbital speed can prevent the onset of pain reducing, in this way, the diameter of the rotation so that diminishing the contact between the crown and the wall of the vessel. The contraindications to the use of OAS atherectomy are the presence of thrombus or dissection, intra-stent or intra-graft stenosis, and caution should be used in vessels with severe tortuosity. In conclusion, OAS represents a safe, efficacious and cost-effective endovascular method for LE-PAD treatment. Its use should be implemented from the correct evaluation of the proper device and from the choose of proper drug treatment to improve the treatment success and to minimize the periprocedural adverse events. Table 2.

### Atherectomy and restenosis: molecular mechanisms and clinical outcomes

This review shows the great improvement in PAD management due to percutaneous treatment. Actually, even if all the techniques discussed below have a good therapeutic impact in terms of regression of LE-PAD as well as in term of days of recovery, they all suffer of an important limitation, the restenosis of the treated area. This drawback is reported in up to 60% of primarily successful PTA [33]. Regardless of the type of intervention, stenosis or restenosis develops in a significant number of patients, often leading to limb loss or death and it remains the “Achille’s heel” of the application of these procedures. Restenosis is arbitrarily defined as a greater than 50% narrowing of vessel diameter compared with a reference artery. The clear mechanism of restenosis is not perfectly known but several evidences suggest a strong link between restenosis itself and vessel inflammation. This theory is feeding the interest toward the identification of makers of inflammations, which may have a diagnostic but especially prognostic role in the managing of the vessels restenosis. The plasma proteins, C-reactive protein (CRP), serum amyloid A (SAA), and fibrinogen are sensitive, specific, and fast reacting markers of acute phase reaction [34] and provide an indirect measure of a cytokine dependent inflammatory process of the arterial wall [35]. Restenosis is mainly due to excessive neointima formation [12]. Percutaneous intervention leads to mechanical injury that induces vascular inflammation, which stimulates vascular smooth muscle cell proliferation and extracellular matrix deposition, resulting in neointimal thickening and restenosis [36]. It has been demonstrated that in a rat model of carotid artery dilation by a balloon catheter, the first step in allowing vascular smooth muscle cell (SMC) proliferation from the tunica media to the intima is the occurrence of internal elastic lamina (IEL) rupture [12]. During arterial catheterization, the endothelial layer is removed by balloon dilation, resulting in the loss of this important anti-thrombogenic layer. Moreover, endothelial denudation results in the exposure of the subendothelial matrix to flowing blood. Platelets and fibrinogen immediately adhere to the surface of the injured vessel, inducing platelets aggregation and activation. Activated platelets release various cytokines, chemokines, and growth factors, which initiate smooth muscle cell (SMC) proliferation and leukocyte recruitment to the injured vessel segment. Substances released or activated after injury include platelet-derived growth factor (PDGF), transforming growth factor (TGF)-β, interleukin (IL)-1, IL-6, IL-8, thrombin, adenosine diphosphate, and thromboxane A2 [33,37]. The initial tethering and rolling of leukocytes on platelets is mediated through binding of the leukocyte receptor P-selectin glycoprotein ligand-1 to platelet P-selectin. Rolling leukocytes stop and firmly attach to adherent platelets when the leukocyte integrin Mac-1 (CD11b/CD18) binds to platelet glycoprotein Ib-alpha or to fibrinogen bound to the platelet glycoprotein IIb/IIIa [36]. After, there is smooth muscle cell (SMC) proliferation and migration to the intima. Migrated smooth muscle cells (SMCs) contribute to the intimal thickening by the excessive synthesis of extracellular matrix (ECM) and proliferation. Intimal SMCs are derived primarily from the media, but they may also be derived from adventitial myofibroblasts, pericytes associated with infiltrating microvessels, and circulating progenitor cells. The pathway for SMC proliferation is an integrated mechanism involving several known and as yet unidentified cell-signalling pathways coupled to the cell cycle. Peptides binding to tyrosine kinase receptors are possibly the most potent mitogens for smooth muscle cells (SMCs) and they modulate a variety of signalling pathways, including ras [38], raf, the mitogen activated protein kinase (MAPK) cascade, the phosphoinositol-3 kinase- protein kinase B pathway and the diacylglycerol protein kinase C pathway [35,39,40]. It was previously described the opposite effects of SMCs proliferation of two intracellular pathways. In fact, it was showed that the stimulation of Ras-MAPKs proteins induces the proliferation of SMCs [38,34] that are, in contrast, inhibited by the activation of cAMP-PKA signaling [41,42]. Furthermore hyperinsulinemia, through activation of the ras–MAPK pathway, rather than hyperglycemia per se, appears to be crucial in determining the exaggerated neointimal response after balloon angioplasty in diabetic animals [43]. Moreover, new growing knowledge about molecular mechanism of restenosis highlight the role of micro-RNA in vascular remodeling [44].

Several studies show the rate of restenosis after baloon angioplasty, after stent implantation and after atherectomy using different types of devices. Ablative therapy deserves particular relevance and several studies have investigated the rates of occurrence of restenosis after debulking procedures. TALON (Treating Peripherals with Silver Hawk Outcome Collections) study involved 601 patients showing a rate of survival free of TLR at 6 months of 90% and at 12 months of 80% [25]. Sarac et al. recruited 167 patients treated with Silver Hawk device in tibial arteries. Cumulative 1-year primary and secondary patency rates were 43% and 57%, respectively [45]. An interesting result comes from Shammas et al. trial which demonstrates the inferiority of Silver Hawk atherectomy versus balloon angioplasty. In this trial 72 patients were divided in two groups. The first group (n=38) were treated with Silver Hawk atherectomy, the second group (n=35) were treated with conventional balloon angioplasty. Primary patency at 2 months was of 34% in the first group versus 56% in the second group [46]. Another kind of device was investigated from Scheiner et al. who studied the impact of Laser ablative atherectomy in SFA arteries. They reported their experience with the excimer laser in recanalizing occluded SFA arteries: the technical success was 90.5%, but primary patency at 1 year was only 33.6%. The 1 year assisted primary and secondary patency rates were 65.1% and 75.9%, respectively. In addition, short SFA occlusions (1–10 cm) treated with the excimer laser demonstrated primary, assisted primary, and secondary patency rates of 49.2%, 76.5%, and 86.3%, respectively, at 36-month follow-up [47]. Stoner et al. reported their data on 40 patients treated with laser atherectomy; average follow-up was 461±49 days. The indication for laser atherectomy was critical limb ischemia in 26 (65%) and claudication in 11 (35%) patients. A total of 47 lesions in the femoropopliteal and infrapopliteal arterial segments were treated. Adjunctive angioplasty was used in 75% of cases. The overall 12 month primary patency was 44% [48]. Two further trials investigated rotational atherectomy: Myers et al. treated 72 patients using rotablator and showed a primary patency of 47% at 6 months, 31% at 12 months and of 18,5% at 24 months [49]. In the OASIS TRIAL, rotational atherectomy with Diamondback 360° was associated with a rate of TLR of 0,9% at 6 months, but further data are needed to understand the impact of this device on long term patency.

### Procedural risks and complications: how to optimize peripheral atherectomy

The risks associated with atherectomy of the superficial femoral, popliteal, anterior tibial, posterior tibial and peroneal arteries may include, but are not limited to the following: arterial dissection, arterial perforation, arterial rupture, arterial spasm, arterio-venous (AV) fistula, bleeding complications, embolism and/or arterial thrombosis, emergency or non-emergency arterial bypass surgery, entry site complications, restenosis of the treated segment, total occlusion of the peripheral artery, vascular complications which may require surgical repair [50,51] . As with any device requiring mechanical deployment and retraction, there exists a risk of mechanical failure of the device resulting in potential surgical intervention. All of the above could cause prolonged illness, permanent impairment of daily function or, in rare cases, death. Possible treatments could include vascular surgery. Extensive reliability engineering testing has been performed on the study device to mitigate risks to the subject due to product failure. Risks of atherectomy may be further limited by providing medications such as aspirin or clopidogrel and continuing to monitor subjects following atherectomy [52,53]. Mechanical atherectomy may be associated with high risk of peripheral embolization. The risk of distal embolization is more common in the acute and subacute peripheral vascular intervention. Embolic protection devices (EPD) have been used successfully in many circumstances. In the femoropopliteal system the size of the common femoral can vary (3-6 mm), then some vessel are too small for most distal protection. The maximum diameter is 5 to 6.3mm depending upon the device used [54]. The use of EPD in acute lower-extremity ischemia is considered a reasonable strategy to prevent distal thrombo-embolism and occlusion of the distal circulation [52]. The embolization rate reported by TALON registry was 0.1% [25]. The use of distal filter has been always associated to retrievement of debris consisting of atherectomy-cut pieces of plaque [55]. In the PROTECT (Preventing Lower Extremity Distal Embolization Using Embolic Filter Protection) registry 40 patients with 56 lesions underwent angioplasty/stenting or atherectomy, with 1 filter employed per patient. Clinically significant macrodebris (>2 mm in diameter) was found in 27.6% of the angioplasty/ stenting patients and 90.9% of the atherectomy patients [56]. Some recommendations to optimize the result of peripheral atherectomy and minimize the risk of procedural complications are listed below: use contralateral access in most cases but, for distal lesions, prefer an antegrade approach for a better control of tip; make slow and methodical cuts and advance the cutting blade slowly; ensure adequate anticoagulation (ACT, activated clotting times of 275 to 300 seconds) to avoid thrombotic complications during the procedure.

## Conclusions

Recent technological advances have made it possible to increase the spectrum of treatable peripheral arterial lesions with high acute procedure success rates. However, the choice of the best endovascular strategy for the treatment of LE-PAD still remains challenging because of the specific features of this vascular bed and the diffuse nature of the atherosclerotic process. Durability and long-term patency remain the major challenges to therapy in LE-PAD endovascular treatment significant advances: despite significant advances and availability of new devices, the principal failure continues to be recurrent restenosis . The role of atherectomy may be to overcome the limitation of balloon angioplasty and stent placement. A number of debulking modalities and devices are now available with good procedural results. Long term outcomes need to be addressed by large, randomized trials.

## List of abbreviations

ABI: ankle-brachial index; AV: arterio-venous; CHD: coronary heart disease; CLI: critical limb ischemia; CRP: C-reactive protein; ECM: extracellular matrix; EPD: embolic protection device; IEL: internal elastic lamina; IL: interleukin; IVUS: intravascular ultrasound; LE-PAD: lower extremity peripheral arterial disease; MAPK: mitogen activated protein kinase; OAS: orbital atherectomy system; PAD: peripheral artery disease; PDGF: platlet-derived growth factor; PTA: percutaneous transluminal angioplasty; PTCA: percutaneous transluminal coronary angioplasty; SAA: serum amyloid A; SAEs: serious adverse events; SFA: superficial femoral arthery; SMC: smooth muscle cell; TGF-β: transforming growth factor; TLR: target lesion revascularization; VH: virtual histology.

## Competing interests

The authors declare that they have no competing interests.

## Authors’ contributions

AF: conception and design, interpetration of data, given final approval of the version to be published, MF, GC, AC, LS, FS RI, RP, SS: acquisition of data, drafting the manuscript, given final approval of the version to be published, BA, BT, CP: critical revision, interpretation of data, given final approval of the version to be published, GE: conception and design, critical revision, given final approval of the version to be published.
